# The Use of Highly Porous 3-D-Printed Titanium Acetabular Cups in Revision Total Hip Arthroplasty: A Systematic Review and Meta-Analysis

**DOI:** 10.3390/jcm14030938

**Published:** 2025-01-31

**Authors:** Peter Richard Almeida, Gavin J. Macpherson, Philip Simpson, Paul Gaston, Nick D. Clement

**Affiliations:** 1 Edinburgh Orthopaedics, Royal Infirmary of Edinburgh, Edinburgh EH16 4SA, UK; 2 Department of Orthopaedics and Trauma, University of Edinburgh, Edinburgh EH8 9YL, UK

**Keywords:** revision total hip arthroplasty, acetabulum, titanium, additive manufacturing

## Abstract

**Background/Objectives**: As the rate of revision total hip arthroplasty (THA) has risen, there has been a drive to improve the technology in the manufacturing of the implants used. One recent advance has been 3-D printing of trabecular titanium implants to improve implant stability and osteointegration. The aim of this study was to review the clinical and radiological outcomes of these acetabular implants in revision THA. **Methods**: A manual search of the databases of the US National Library of medicine (PubMed/MEDLINE), Embase, and the Cochrane library was performed. The following keywords of “revision total hip arthroplasty” AND “acetabulum” AND “titanium” AND “porous” were utilised. **Results**: In total, 106 abstracts were identified during manual search of databases. In total, 11 studies reporting 4 different implants, with a total of 597 operated joints in 585 patients were included in this review. There were 349 (59.7%) female patients, and the mean age per study ranged from 56.0 to 78.4 years. The all-cause survival rate was 95.52% (95% CI: 92.37–97.96) at a mean follow up of 3.8 years (95% CI: 2.6–5.1). The 40 cases that required re-operation included 17 (2.8%) for infection, 14 (2.3%) instability, 2 (0.3%) for shell migration and 5 (0.8%) for aseptic loosening. The most commonly used patient reported outcome measure was the Harris Hip Score with the mean post-operative score of 86.7 (95% CI: 84.3–89.1). **Conclusions**: Trabecular titanium acetabular implants, when used in revision THA, resulted in excellent short- to mid-term outcomes with improved hip specific outcomes and a survivorship of 95.52% over the reported follow-up period. Future prospective studies evaluating long term outcomes are needed to make comparisons between more established solutions used in revision THA.

## 1. Introduction

Over one million total hip arthroplasty (THA) procedures are performed annually worldwide, and the procedure is regarded as one of the most successful orthopaedic interventions [1,2]. THA is a cost-effective treatment for end-stage osteoarthritis of the hip with the majority of patients enjoying improvement in pain, hip function and health related quality of life within the first year after surgery [3]. The annual number of THAs performed is predicted to increase by 176% by the year 2040 and 659% by the year 2060 in the United States of America alone [4,5,6]. More than half of primary THA will survive past 25 years, leaving many requiring revisions during the lifetime of the recipient [7]. In keeping with rising numbers of primary THA there is a parallel increase in the number of revision THAs [3,8]. In the United States alone, revision THA grew 36% between 2002 and 2014 to a total of 50,220 procedures annually, with a further forecasted growth of 42% by 2040 and 101% by 2060 [3,8]. Data from the National Joint Registry in England and Wales reported that on average 4.3% of primary THA will require revision within 10 years [7]. Further analysis showed that of these revisions 10.8% will require a second revision and 1.8% require a third revision [9].

THA are commonly revised for periprosthetic joint infection (PJI), aseptic loosening, instability, peri-prosthetic fracture, and adverse reactions to particulate debris [7,10,11]. Key challenges that need to be overcome in revision THA include establishing stability and restoring hip biomechanics in the presence of bone loss and poor bone quality [10,12]. The rising volume of revision surgery has driven technological advancements in modern implants to both reduce the revision rate of primary THA as well as providing solutions to perform more complex revision THA. First generation designs of uncemented acetabular cups have proven to perform well, with 70.1% to 89.3% survivability at 15 years; however, aseptic loosening remains a common reason for failure [13,14]. With conventional manufacturing methods in uncemented cups, various techniques have been utilised to enhance initial and long-term component stability such as titanium plasma spray, grit blasting, cobalt chrome beads, titanium metal fibre, and hydroxyapatite [15,16].

Over the last decade ‘additive manufacturing’ has grown in use in manufacturing 3-dimensional (3-D)-printed ultra porous titanium acetabular cups in bulk with a porosity of >60% and mean pore size > 400 nm while maintaining the advantages of titanium [15]. These cups are effectively produced from trabecular titanium [17]. Their characteristics have been proposed to provide increased stability compared to conventional manufacturing methods due to their high porosity, higher coefficient of friction against bone, and a modulus of elasticity nearer to that of bone [12,15,18]. These characteristics of 3-D-printed trabecular titanium components differ to conventionally manufactured components which utilise computer numerical controlled (CNC) machining to create a dense solid dome with various coated surfaces to increase porosity [19].

Additive manufacturing has been proposed as an improvement to conventionally manufactured acetabular implants; however, it is still a relatively new technology with notable differences amongst 3-D-printed titanium acetabular cups currently available. The purpose of this review was to investigate the short- to mid-term clinical and radiographic outcomes of revision THA with ultra porous titanium cups produced with 3-D printing additive manufacturing technology.

## 2. Materials and Methods

### 2.1. Search Criteria

The Preferred Reporting Items for Systematic reviews and meta-analyses (PRISMA) criteria was followed to conduct this systematic review [20]. A manual search was performed of the databases of the US National Library of medicine (PubMed/MEDLINE), Embase, and the Cochrane library. The following keywords of “revision total hip arthroplasty” AND “acetabulum” AND “titanium” AND “porous” were utilised. The study protocol was registered in the International Prospective Register of Systematic Reviews (PROSPERO): study number CRD42024565355.

### 2.2. Inclusion Criteria and Exclusion Criteria

The inclusion criteria were clinical trials investigating revision total hip arthroplasty using off the shelf highly porous titanium acetabular implants manufactured with 3-D printing additive technology. Highly porous was defined as porosity > 60% and >400 microns mean pore size [15].

The exclusion criteria were (1) non-English language studies, (2) studies with less than 10 participants, (3) studies without radiological, clinical or functional outcomes reported, (4) biomechanical studies, (5) reviews or systematic reviews, (6) studies assessing use of cages, cup cages, oblong cups, tantalum metal, or customised implants, (7) studies with less than 2 years mean follow up, (8) non-full-text articles.

### 2.3. Data Collection

Two authors searched the relevant databases independently and compiled a list of studies matching the inclusion and exclusion criteria. From the studies that were included the following information was tabulated: title, author, year of publication, study design, number of patients/joints, gender, age, BMI, type of acetabular component, classification of acetabular defect, clinical outcomes, radiological outcomes, reason for revision, and complications.

### 2.4. Assessment of Study Quality

The Methodological Index for Non-Randomized Studies (MINORS) score was used in the assessment of the quality of the studies included. The MINORS score is useful when assessing non-randomized studies, and has been used in many arthroplasty studies. It consists of 8 questions that can be scored individually 0 to 2 [21]. The question is scored as 0 if the relevant information is not included in the study, 1 if it is included but not adequately described and 2 if it is included and described well [21]. The study is graded as poor if the score is lower than 5, moderate with a score of 6–10, and good if the score is 11–16 points. Two authors calculated the scores, with a third author consulted if there was any disagreement (Table 1).

### 2.5. Statistical Analysis

Continuous variables were presented with range or mean values and standard deviation. Categorical variables were presented with frequency and percentages. Statistical analysis was performed using RStudio Version 4.4.2 (R Foundation for Statistical Computing, Austria). Heterogeneity was assessed using study mean age, sex, follow-up, duration, and implant type using I^2^, with I^2^ > 50% considered heterogenous, where random effects methods were preferred. Due to residual heterogeneity (I = 53.46% [95% CI: 8.4–76.3], *p* = 0.090) random effects meta-analysis was used to determine effect size estimates for overall survival and postoperative Harris Hip Score (HHS), weighted by sample size. HHS standard deviation data were approximated from median and range using established techniques in one study [22], and through imputation in one study. The Freeman–Tukey double arcsine transformation was used for all meta-analyses and data were back-transformed prior to interpretation. Effect sizes with 95% confidence intervals were reported for all analyses.

## 3. Results

### 3.1. Search Results

**Figure 1:**
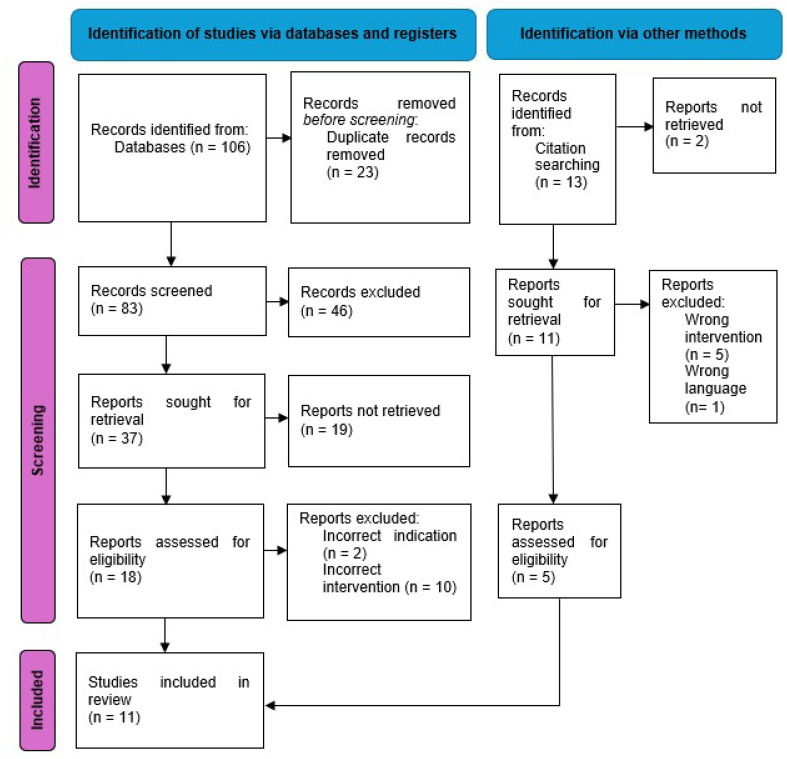
Flow chart of study selection according to PRISMA guidelines [20].

Using the keywords described above resulted in the identification of 106 abstracts. (76 in PubMed, 23 in Embase and 7 in Cochrane) (Figure 1). Duplicate articles were identified and removed. The remaining abstracts were screened using the inclusion and exclusion criteria. In total, 18 articles were subjected to further analysis with review of the full text, with a total of 6 articles that fulfilled all criteria and were included in the review. An additional five articles were identified through the citation process that matched the inclusion and exclusion criteria and were therefore included. The level of evidence for all studies was level three with the exception of one case series (Table 1).

**Table 1:** Type of study, level of evidence and modified Coleman score.

Authors	Type of Study	Quality of Evidence	MINORS Score
Castagnini et al. (2021) [23]	retrospective case series	IV	11
Cozzi Lepri et al. (2022) [10]	retrospective cohort	III	9
De Meo et al. (2018) [11]	retrospective cohort	III	11
Shaarani et al. (2023) [12]	retrospective cohort	III	10
Shang et al. (2022) [24]	retrospective cohort	III	11
Shichman (2022) [25]	retrospective cohort	III	10
El Ghazawy et al. (2022) [26]	retrospective cohort	III	10
Perticarini et al. (2021) [27]	retrospective cohort	III	10
Munegato et al. (2018) [28]	retrospective cohort	III	10
Gallart et al. (2016) [29]	retrospective cohort	III	10
Steno et al. (2015) [17]	retrospective cohort	III	11

### 3.2. Demographics

In total, 585 patients with 597 operated joints were included in this review. There were 349 (59.7%) female patients. The mean age per study ranged from 56.0 to 78.4 years. The mean body mass index varied between 25.61 and 30.36 kg/m^2^. The mean reported follow-up periods ranged between 25.7 months and 91 months (Table 2).

### 3.3. Indications for Surgery

The most common indications reported for revision surgery were aseptic loosening with 355 cases (59.5%), periprosthetic joint infection (PJI) with 77 cases (12.9%) and instability with 62 cases (10.4%). Other causes included symptomatic elevation of metal ions in metal-on-metal bearing surface, osteolysis, pseudotumour/metalosis, trunnionosis, implant failure and periprosthetic fractures (Table 3).

### 3.4. Classification of Acetabular Defects

All studies in this review used the Paprosky Classification when describing acetabular defects (Table 3). Paprosky Type I included 69 cases (11.6%), Paprosky Type II 71 cases (11.9%), Paprosky Type IIA 52 cases (8.7%), Paprosky Type IIB 108 cases (18.1%), Paprosky Type IIC 75 (12.6%), Paprosky Type III 46 cases (7.7%), Paprosky Type IIIA 92 cases (15.4%), Paprosky Type IIIB 82 cases (13.7%), and Paprosky Type IIIC 2 cases (0.3%).

### 3.5. Type of Implants

In total, 4 different acetabular systems were reported in the 11 studies (Table 4). In total, 7 studies reported on the use of the Delta (Trabecular Titanium) TT system (Limo Corporate, San Daniele, Italy), including 19 (3.2%) Delta TT, 228 (38.2%) Delta One TT and 210 (35.2%) Delta Revision TT with a total of 457 (76.5%) joints [10,11,17,26,27,28,29]. Two studies reported on the REDAPT shell (Smith and Nephew, Memphis, USA) with a total of 99 joints (16.6%) [12,25], one study reported on the Ti-por cup (Adler Ortho, Milan, Italy) with 18 joints (3.0%) [23] and one study reported on the Aikang TT cup (Beijing, China) in 23 joints (3.9%) [24].

A total of 6 studies included information regarding bearing surfaces and head size [10,12,25], with 1 study using dual mobility in all 85 revision cases [10], and 4 other studies employed dual mobility for a proportion of their cohort. Dual mobility was used in a total of 128 cases (21.4%). All studies, with the exception of one, reported on the use of allograft (*n* = 319, 53.4%), eight studies used augments (n = 84, 14.1%) and two studies used medial wall mesh (n = 16, 2.7%) [10]. Only seven studies reported on the use of screws for cup stabilisation [10,12,23,25].

**Table 4:** Description of implants, cups, bearings, augments, grafts and screws.

Authors	Manufacturer of Implant	Type of Acetabular Cup	Details of Acetabular Cup Size	Bearing Surfaces	Use of Grafts and Augments	Use of Screws
Castagnini et al. (2021) [23]	Adler	Ti-por cup	50.6 mm ± 3.6 (range 46–56) (Mean)	not specified	3 (16.7%) morselise bone allograft	3 cases (16.7%)
Cozzi Lepri et al. (2022) [10]	Lima	Delta TT one in 30 (35.3%), Delta TT revision in 55 (64.7%)	not specified	85 (100%) Dual mobility	85 (100%), corticocancellous allograft, 12 (14.11%) medial wall meshes	4.2 (range 2–7) (Mean)
De Meo et al. (2018) [11]	Lima	Delta TT one in 39 (60.9%), Delta TT revision in 25 (39.1%)	not specified	not specified	34 (53.1%), morselise bone allograft, 4 (6.3%) augment	not specified
Shaarani et al. (2023) [12]	Smith and Nephew	REDAPT shell	54 mm (Mode)	29 (49.15%) Dual mobility	6 (10.17%) augment	4 (Median)
Shang et al. (2022) [24]	Aikang corp	Aikang TT	not specified	not specified	4 (17.39%) augment, 1 (4.35%) structural bone graft, 5 (21.74%) augment and bone graft	not specified
Shichman (2022) [25]	Smith and Nephew	REDAPT shell	60 mm (range 48–80) (Median)	36 mm (range 28–36) (Median)	12 (30%) Allograft	4 (range 2–8) (Median)
El Ghazawy et al. (2022) [26]	Lima	Delta TT revision	Not stated	Head size not stated, 3 (12.5%) Dual mobility	20 (83%) Morselised allograft, 19 (80%) augments	3 (range 2–4) (mean)
Perticarini et al. (2021) [27]	Lima	Delta revision TT in 39 (41.1%), Delta One TT in 56 (58.9%)	46–66 mm	6 (6.3%) Dual mobility	57 (60%) Allograft, 13 (13.7%) augments, 4 (4.2%) meshes	2–6 screws (range)
Munegato et al. (2018) [28]	Lima	Delta TT revision	Not stated	Not stated	24 (66.7%) Allograft, 11 (30.6%) synthetic bone graft	not stated
Gallart et al. (2016) [29]	Lima	Delta Revision TT 18 (25%), Delta One TT 54 (75%)	Not stated	Not stated	22 (30.6%) Allograft, 17 (23.6%) augment	not stated
Steno et al. (2015) [17]	Lima	Delta TT 19 (23.5%), Delta One TT 49 (60.5%), Delta Revision TT 13 (16%)	Mode 58 (range 44–68)	5 (6.2%) Dual mobility	53 (65.4%) Morselised allograft, 3 (3.7%) structural allograft, 16 (19.8%) augments	2–5 screws (range)

### 3.6. Clinical Outcomes

All patient reported outcome measures improved postoperatively in all studies (Table 5). The most common reported clinical outcome measure reported was the HHS, which was used in eight studies (n = 385, 64.5%) [10,11,23,24,25,26,27,28]. Meta-analysis of HHS was calculated as a mean of 86.7 (95% CI: 84.3–89.1). One study used the Short-form 36 (SF-36) (mean 754.04, standard deviation [SD] 22.74) and the Visual Analogue Scale (VAS) (1.14 SD 0.23) in combination with HHS [24], and another study used length of stay (LOS) (5.34 days ± 3.34) [25]. One study used the Oxford hip score (OHS) (83, SD 15) and Short-form 12 (SF-12) physical (44, SD 11), SF-12 mental (56 SD 10), Western Ontario and McMaster Universities Osteoarthritis Index (WOMAC) (85 SD 17) [12]. Two studies used Merle d’Aubigné-Postel scores [17,29].

### 3.7. Imaging Outcomes

All studies reported on radiological outcomes. One study used the criteria according to Moore et al. (2006) to assess osteointegration [23,30]. Two studies used Gie et al. (1993) to report on bone graft incorporation [10,28,31]. Four studies reported no radiolucencies on post-operative radiographs at follow-up, and two studies reported six (1.0%) cup migrations (Table 5).

### 3.8. Survival and Complication Rates

Meta-analysis of all-cause cup survival was 95.52% (95% CI: 92.37–97.96) at a mean follow up of 3.8 years (95% CI: 2.6–5.1). In total, 80 (13.4%) complications were reported with 40 (6.7%) complications not requiring re-operation that included deep vein thrombosis (DVT) (n = 20, 3.4%), wound problems (n = 3, 0.5%), instability (n = 3, 0.5%), cup migration (n = 4, 0.7%), heterotrophic ossification (n = 3, 0.5%), femoral neurapraxia (n = 4, 0.7%), psoas tendinopathy (n = 1, 0.2%), persistent pain (n = 1, 0.2%), and trochanteric bursitis (n = 1, 0.2%). In total, 40 cases (6.7%) required re-operation with causes including 17 (2.8%) cases of PJI, 14 (2.3%) cases of instability, 5 (0.8%) cases of aseptic loosening and 2 (0.3%) cases of shell migration (Table 5).

**Table 5:** Clinical and radiological outcome measures, survival rate and complications.

Authors	Clinical Outcomes	Radiological Outcomes	Survival Rate	Complications	Causes for Re-Operation
Castagnini et al. (2021) [23]	HHS 88.3 ± 9.2 (range 68–97)	No cup loosening or cup migration at final follow up. No periacetabular radiolucency. Good cup osseointegration according to Moore et al. (2006) [30] of >3 in every case.	100%	4 complications in 3 (16.7%) cases. 1 (5.6%) PJI, 1 (5.6%) wound infection, 1 (5.6%) dislocation, 1 (5.6%) psoas tendonitis	No revision
Cozzi Lepri et al. (2022) [10]	HHS 89.7 (range 83–98)	Bone graft incorporation according to Gie et al. (1993) [31] type 1 (n = 8, 9.4%), type 2 (n = 22, 25.9%), type 3 (n = 55, 64.7%).	5 year 100%, 10 year 88%	19 (22.4%) DVT, 4 (4.7%) femoral neuropraxia, 1 (1.2%) aseptic loosening, 1 (1.2%) PJI	2 (2.3%) cases re-operation after 5.6 years. 1 (1.2%) PJI, 1 (1.2%) aseptic loosening
De Meo et al. (2018) [11]	HHS 83.7 (range 58.9–91.3)	No radiolucent lines or signs of migration were observed.	Kaplan–Meier survivorship curve at 48.3 months showed survivorship of 89.7% for revision and 94.8% for acetabular cup removal	3 (5.2%) instability, 2 (3.4%) PJI, 1 (1.7%) aseptic loosening	6 (10.3%) cases re-operation. 3 (5.2%) instability, 2 (3.4%) PJI (3.4%), 1 (1.7%) aseptic loosening
Shaarani et al. (2023) [12]	OHS 83 (SD 15), SF-12 physical 44 (SD 11), SF-12 mental 56 (SD 10), WOMAC function score 84 (SD 17), WOMAC stiffness score 83 (SD 15), WOMAC pain score 85 (SD 15), WOMAC global score 85 (SD 17)	2 (3.4%) Shell migration, no radiographs demonstrated radiolucency.	-	2 (3.4%) shell migrations, 1 (1.7%) acute PJI	1 (1.7%) revision of liner for PJI. 1 (1.7%) planned revision of cup migration
Shang et al. (2022) [24]	HHS 90.48 SD 3.65, SF-36 754.04 SD 22.74, VAS 1.14 SD 0.23	All cups remained stable with no loosening and no changes in cup abduction angle. According to bone growth criteria from Anderson Orthopaedic Research institute, 2 cups had 2 signs, 17 had 3 signs, 4 had 4 signs.	100%	1 (4.3%) persistent pain, 1 (4.3%) persistent wound drainage	No revisions
Shichman (2022) [25]	HHS 83.53 ± 12.15, LOS 5.34 ± 3.34,	39/40 (97.5%) cups had osteointegration, 1 (2.5%) reported cup migration.	Kaplan-Meier showed all-cause revision free survival rate of 95.0% at 6 months and 1 year, and 92.0% at 4 years	2 (5%) Acute PJI, 1 (2.5%) implant migration with aspetic loosening, 1 (2.5%) DVT	1 (2.5%) Implant migration with aspetic loosening, 2 (5%) PJI
El Ghazawy et al. (2022) [26]	HHS 85 (range 70–98)	No change in cup position. No progressive radiolucency.	100%	No complications	No re-operations
Perticarini et al. (2021) [27]	HHS 84.4 (range 46–99) SD 7.56	1 (1.1%) graft resorption. All other cups no cup migration or aseptic loosening.	88.54% (95 CI 80.18–93.52%) at 71 months	7 (7.3%) PJI, 7 (7.3%) Instability, 1(1.1%) graft resorption with aseptic loosening, 2 (2.1%) periprosthetic femur fracture, 1 (1.1%) trochanteric bursitis, 3 (3.2%) heterotrophic ossification	7 (7.3%) PJI, 5 (5.3%) instability, 1 (1.1%) graft resorption with aseptic loosening, 2 (2.1%) periprosthetic femur fracture
Munegato et al. (2018) [28]	HHS 87 (SD ± 7.7)	No signs of loosening, bone graft graded to Gie: 21 (58.3%) Type 3, 12 (33.3%) Type 2, 2 (5.6%) Type 1.	100% for aseptic loosening, 91.7% for any revision	1 (2.8%) PJI, 2 (5.6%) instability	1 (2.8%) PJI with dislocation, 2 (5.6%) cases of instability that developed PJI after re-operation
Gallart et al. (2016) [29]	Merle d’Aubigné-Postel score pain 5.7 ± 0.7, walking 5.3 ± 0.7, range of motion 5.6 ± 0.7	Not stated.	88.89%	3 (4.2%) PJI, 3 (4.2%) Instability, 2 (2.8) aseptic loosening	3 (4.2%) PJI, 3 (4.2%) Instability, 2 (2.8) aseptic loosening
Steno et al. (2015) [17]	Merle d’Aubigné-Postel functional score 9.78, pain 5.45 (range 3–6), walking 4.33 (range 3–6)	3 (3.7%) initial cup migrations that stabilised with no radiolucency at final follow up.	98.77%	3 (3.7%) cups with medial migration that stabilised, 1 (1.2%) instability	1 (1.2%) instability

## 4. Discussion

When compared to primary THA, revision THA is associated with an increased risk of complications and places increased physiological, psychological and economic burdens on both the patient, healthcare providers and the healthcare system [24,32]. Revision surgery is complex and is fraught with technical difficulties in dealing with poor bone quality and bone defects. Acetabular components are more frequently revised than stems in isolated component revisions [33]. The overall success rates for revision THA ranges from 61.3% to 98.3% [34]. Uncemented implants have shown better success rates compared to cemented components in revision THA [17]. Finding the balance between stable acetabular cup fixation and restoration of the hip biomechanical parameters is necessary for improved success in revision THA [10]. Porous metals have been the most recent advance in surface technology, with creation of 3-D structures of interconnected porous channels similar to trabecular bone to achieve this balance [16]. Initially, tantalum was used in the manufacturing of these highly porous components of trabecular metal with well-established favourable short- and mid-term survival [16,27,35]. However, tantalum is a rare metal with far less available for 3-D printing applications compared to titanium due to technical and economic factors [36]. Additive manufacturing using 3-D printing techniques, with a –aluminium–vanadium (Ti-6Al-4 V) alloy powder, has evolved from traditional manufacturing methods of “formative shaping” or “subtractive manufacturing” using titanium with various techniques of coating [24].

Additive manufacturing results in up to 75% reduction in raw material usage and up to 50% reduction in the costs of the manufacturing process [37]. Additive manufacturing combines the beneficial properties of titanium, including biocompatibility, strength and resistance to corrosion, with the benefits of high porosity components in bulk production of readily available implants [13,14,32]. Three-dimensional printing techniques may use Electron Beam Melting (EBM) or Selective Laser Melting (SLM) to produce highly porous components with specific pore size, shape and density [11,24]. This effectively results in titanium trabecular metal implants, which have technical parameters that are similar to those of more established tantalum implants with mean pore size of 550 μm and porosity of 75–80% [10,35]. Highly porous titanium acetabular shells have elasticity and a micro-structure similar to bone, improving the initial and long-term stability with a high co-efficient of friction and a structure promoting osseointegration [13,25].

There are a growing number of manufacturers using additive manufacturing technology in creating acetabular components (Table 6). The use of ‘off the shelf’ highly porous 3-D-printed titanium acetabular shells for revision arthroplasty surgery has been evaluated in only a few studies. This current review provides evidence that highly porous 3-D-printed titanium acetabular shells resulted in good early to mid-term outcomes when employed for revision THA, with an all-cause survivorship of 95.52%, with four studies reporting 100% survivorship at mean follow-up of 1.7 years, 3.5 years, 5 years and 5.7 years.

It is difficult to make direct comparisons between studies reporting the outcomes of revision THA due to the heterogenicity of indications, varying morphology of bone defects and the combinations of implant usage available [25]. Vutescu et al. (2017) made adjustments for acetabular defect severity when comparing more established trabecular tantalum to ultra porous titanium implants used in revision THA, and found no difference, with both implants resulting in excellent outcomes at 5 years [16]. Previous systematic reviews have assessed the outcomes of 3-D-printed titanium cups; however, to the authors knowledge, this is the first review assessing the use of different types of 3-D-printed titanium implants for revision THA. The majority of the implants reported in this review used the Delta TT system (Lima corporate, San Daniele, Italy). This system has been available since 2007, giving it the longest period of availability for use [29].

Cacciola et al. (2023) previously reviewed 3-D-printed titanium acetabular implants and reported an overall survival rate of 93.4%, similar to the findings of this study of 95.52% [39]. They included a total of 523 hip revisions from eight studies; however, they only included Delta TT systems (Lima corporate) in their review [39]. Instability was the most common complication of 4.1%, with aseptic loosening of 1.5%, which differs from the results of the current review, with PJI being the most common complication followed by instability requiring re-operation. The majority of the studies from Cacciola et al. (2023) were included in the current systematic review, which also included the results from the Delta Revision TT cup [39]. This revision cup is 3-D printed using EBM but differs from Delta One TT and Delta TT cups, and all other hemispherical titanium cups, due to a “cage construct” with a built-in hook and three ‘winglets’ [17]. Whether these have a significant clinical impact on implant survival is still to be determined, although most studies report their use in more severe defects which may bias outcomes. Gallart et al. reported no difference (*p* = 0.101) in failures of aseptic loosening with Delta One cups compared to Delta TT Revision components [29]. Future studies comparing these cups to standard hemispherical designs as well as other trabecular metal designs are needed.

The systematic review by Malahias et al. (2019) assessed highly porous titanium acetabular cups in both primary and revision settings. It was reported that in revision THA, there was an overall acetabular revision rate of 6.5% [15]. The rates of aseptic loosening of the acetabular component (2.4%), PJI (2.4%) and dislocations (2.4%) were also found to be low and comparable to the findings of this current review [15]. There was improvement in all clinical scores [15]. Although the inclusion criteria were specific with mean porosity and mean pore size similar to components manufactured with 3-D printing, the study also included ultra-high porosity implants that were not 3-D printed with additive technology such as the Trident acetabular cup (Stryker), the predecessor to the Trident II acetabular cup (Stryker, Mahwah, USA) [15]. These are highly porous titanium acetabular cups used in revision THA and have shown survivorship ranging from 91% to 98.4% after at least 5 years [40,41,42]. Although the cups reported had similar surface characteristics of mean pore size and pore density, they are manufactured with conventional techniques and lack the proposed advantages of 3-D-printed trabecular titanium [15].

Tsikandylakis et al. (2020) reported their randomised control trial comparing 3-D-printed titanium cups to cups conventionally manufactured with porous plasma spray (PPS) [33]. It was found that 3-D-printed cups were not superior in cup fixation within the 2-year follow-up period in respect to radiological and clinical outcomes, but only primary THA cases were assessed [33]. In this current review, only one study compared 3-D-printed cups to non-3-D-printed cups used in revision THA, which demonstrated that 3-D-printed cups had better HHS and SF-36 scores and had significantly better bone ingrowth than conventionally manufactured cups [24]. The difference in findings between these two studies may be due to the difference of comparing primary versus revision THA, and that the benefits of 3-D-printed cups are more pronounced in settings of compromised bone quality and quantity. This is similar to the difference found between trabecular metal tantalum cups and hydroxyapatite-coated titanium cups by Meneghini et al., with a more pronounced difference in outcomes noted in major bone deficiency compared to minor defects [17,43]. The systematic review with meta-analysis by Shen et al. (2022) found a survival rate of 92.5% at 10-year follow up for tantalum cups, and when comparing tantalum to titanium implants, it was found tantalum acetabular cups had fewer complications of aseptic loosening and PJI, but more dislocations compared to titanium cups [44]. These cups were not 3-D printed [44]. There are obviously differences noted between all these implants. More studies directly comparing 3-D printed titanium implants to conventionally manufactured titanium implants to, as well as to tantalum implants used in revision THA are needed.

Only six studies reported on the type of bearing surface and five studies reported on cup size; however, the parameters used were inconsistent, making comparisons difficult. One reported advantage of 3-D-printed titanium acetabular cups is the cup size optimisation due to the thinner implant thickness of these components [14]. Information regarding the cup size, polyethylene liner size and head size as well as the bearing surface used may be beneficial to include in future studies to determine the clinical significance of these parameters.

All studies in this review reported on the use of augments and bone grafts, but only seven studies reported on the use of screws. Shaarani et al. (2023) found that there was an increase in the quantity of screws with increased age [12]. This relationship may suggest that there were increased screws in the presence of decreased bone quality; however, since bone quality is difficult to objectively quantify this proposed relationship cannot be confirmed. The details of the types of graft used were not always included, although Strahl et al. (2023) found no significant difference in success rates between the use of different allografts, including bulk structural grafts and morselised grafts in a recent systematic review [34]. These findings need to be interpreted with caution as the type of bone graft used is largely dictated by the morphology of the bone defect present.

Shaarani et al. (2023) reported a negative correlation between cup size and augments [12]. This suggests that large cups, including ‘jumbo cups’ were used to adequately address bone defects without the use of augments; however, this was found to be at the expense of raising the hip centre of rotation [12]. Unfortunately, none of the studies examined the impact of bone graft, augments, screws, cup size and bearing surfaces on clinical outcomes and incidence of complications. The difficulty of this is not underestimated, as the usage of bone grafts, augments and screws usually indirectly indicates the complexity of the case with the quantity and quality of bone available. Further studies evaluating the combinations of these components used with 3-D-printed titanium cups in revision THA setting are needed.

Contact between host bone and the implant surface is crucial for the success of acetabular components in revision THA [29]. The necessary surface area contact may differ between implants depending on the qualities of the implant including biocompatibility, strength, elasticity, and mean pore size and porosity [10,32]. Bone loss was classified according to Paprosky by all studies in this review, with Paprosky type IIB being the most common pattern of bone loss reported, followed by Type IIIA, Type IIIB and Type IIC. Outcomes and failures were not matched according to bone loss defects in the majority of studies.

Gallart et al. compared acetabular component failures according to the Paprosky classification. Paprosky type 1 had no failures, and more severe bone defects of Paproksy type II and type III had four failures each (*p* = 0.028) [29]. When interpreting these results, it should be noted that allograft or augments were used in the majority of Paprosky Type II defects whilst Delta Revision TT cups with built in flanges were used in Paprosky type III [29].

Steno et al. (2015) reported no acetabular cup migrations except for three cases in which Delta Revision TT was combined with allograft in Paprosky type IIIB defects, with all other defects having 100% survival and no cup migrations [17]. Comparing outcomes to the bone defects and the type of implants used in future larger studies may be beneficial in providing insights into the limit of usage of different implants.

The limitations of this review relate to the quality of the studies being examined. There are no level 1 or level 2 data available, and the use of inclusion/exclusion criteria is variable. The difference in study designs, patient populations and follow-up periods makes comparative research difficult. There were no prospective studies available and average values, SD and ranges were reported inconsistently by the various included studies. The use of different outcome measures in both clinical assessment and radiological assessment made comparative research challenging.

## 5. Conclusions

Revision THA are complex procedures often with diminished bone quality and quantity available for component fixation. The evolution of a new generation of titanium acetabular implants using 3-D printing additive manufacturing techniques have resulted in excellent short- to mid-term outcomes in this review. Future prospective long-term studies, with standardization of both clinical and radiological assessment tools, are needed to compare the success and longevity of these implants to other more established options such as tantalum trabecular metal and readily available conventionally manufactured titanium implants.

## Figures and Tables

**Table 2:** Summary of demographic details.

Authors	Number of Hips (Patients)	Gender F	Gender M	Mean Age	Mean BMI (kg/m^2^)	Mean Follow Up
Castagnini et al. (2021) [23]	18 (16)	13	3	62.3 ± 8.3 (range 51–83)	26.2 ± 3.1 (range 21.4–31.2)	5.7 years ± 0.7 (range 5–7 years)
Cozzi Lepri et al. (2022) [10]	85	50	35	67.8 (range 32–83)	26.9 (95% confidence interval 25.4–27.7, range 18.3–33)	6.12 years (range 2–10.2)
De Meo et al. (2018) [11]	64	37	27	78.4 (range 42–87)	26.1 (range 23.5–33.2)	48.3 months (range 38–82.3)
Shaarani et al. (2023) [12]	59 (55)	34	25	68.8 SD 12.3	26.6 SD 5.9	25.7 months SD 13.8 (range 4–52)
Shang et al. (2022) [24]	23	13	10	70.35 ± 8.1	25.61 ± 2.80	41.82 months ±11.44 (range 24–64)
Shichman (2022) [25]	40	22	18	71.42 ± 9.97	30.36 ± 6.88	2.21 years ±0.77
El Ghazawy et al. (2022) [26]	24	6	18	56 (range 30–67)	Not stated	20.75 months (14–30)
Perticarini et al. (2021) [27]	95	65	30	70 (range 29–90) SD 11	25.68 (range 17–36.67) SD 3.7	91 months (24–146)
Munegato et al. (2018) [28]	36 (34)	24	14	75 (range 45–92)	Not stated	39.8 months (12–91.5)
Gallart et al. (2016) [29]	72 (69)	34	38	70.7 SD 10.3	Not stated	30.5 months SD 16.9
Steno et al. (2015) [17]	81 (80)	51	30	68.3 (range 32–84)	Not stated	38.14 months (24–62)

SD: Standard deviation.

**Table 3:** Type of acetabular defects and indications for surgery.

Authors	Acetabular Defects	Indication for Surgery
Castagnini et al. (2021) [23]	Paprosky I 15 (83.3%), Paprosky II 3 (16.7%)	All cases were revisions of Du Puy ASR XL metal on metal bearing surface. 5 (27.8%) aseptic loosening and raised metal ions, 8 (44.4%) pain and metal ions over threshold, 4 (22.2%) osteolysis, 1 (5.6%) pseudotumour.
Cozzi Lepri et al. (2022) [10]	Paprosky IIB 23 (27.1%), Paprosky IIC 20 (23.5%), Paprosky IIIA 24 (28.2%), Paprosky IIIB 18 (21.2%)	31 (36.5%) aseptic loosening, 19 (22.3%) recurrent instability, 15 (17.6%) adverse reaction to metal debris (ARMD), 11 (13%) PJI, 9 (10.6%) periprosthetic fracture.
De Meo et al. (2018) [11]	Paprosky IIB 25 (39%), Paprosky IIC 15 (23.4%), Paprosky IIIA 15 (23.4%), Paprosky IIIB 9 (14.1%)	28 (43.75%) aseptic loosening, 26 (40.6%) instability, 10 (15.6%) wear debris osteolysis.
Shaarani et al. (2023) [12]	paprosky I 21 (35.6%), Paprosky IIA 19 (32.2%), Paprosky IIB 3 (5.1%), Paprosky IIC 9 (15.3%), Paprosky IIIA 4 (6.8%), Paprosky IIIB 3 (5.1%)	21 (35.59%) aseptic loosening, 11 (18.64%) PJI, 3 (5.08%) instability, 3 (5.08%) failed DHS, 5 (%) failed hip resurfacing, 2 (3.39%) metastatic disease, 1 (1.69%) acetabular erosion from hemi arthroplasty, 1 (1.69%) squeaking ceramic on ceramic, 1 (1.69%) native hip joint dislocation, 1 (1.69%) broken cement/osteolysis, 1 (1.69%) neck of femur fracture, 1.69%) acetabular fracture, 8 (13.56%) peri-prosthetic fracture, 1 (1.69%) stem fracture.
Shang et al. (2022) [24]	Paprosky I 4 (17.39%), Paprosky II 15 (65.22%), Paprosky III 4 (17.39%)	17 (73.91%) aseptic loosening, 6 (26.09%) PJI.
Shichman (2022) [25]	Paprosky I 1 (2.5%), Paprosky IIA 10 (25%), Paprosky IIB 14 (35.0%), Paprosky IIC 2 (5%), Paprosky IIIB 11 (35%), Paprosky IIIC 2 (5%)	22 (55%) aseptic loosening, 8 (32%) PJI, 2 (5%) instability, 1 (2.5%) trunnionosis, 1 (2.5%) pseudotumour, 6 (15%) complex primary.
El Ghazawy et al. (2022) [26]	Paprosky IIIA 7 (29.2%), Paprosky IIIB 15 (62.5%), Paprosky IIB 2 (8.3%)	19 (79.2%) aseptic loosening, 3 (12.5%) PJI, 2 (8.3%) revision hemiarthroplasty for acetabular erosion.
Perticarini et al. (2021) [27]	Paprosky II 53 (55.8%), Paprosky III 42 (44.2%)	86 (82.69%) aseptic loosening, 8 (7.69%) metallosis, 4 (3.85%) periprosthetic fracture, 3 (2.88%) implant failure, 2 (1.92%) instability, 1 (0.96%) PJI.
Munegato et al. (2018) [28]	Paprosky IIB 5 (13.9%), Paprosky IIC 7 (19.4%), Paprosky IIIA 15 (41.7%), Paprosky IIIB 9 (25%)	33 (91.7%) aseptic loosening, 2 (5.6%) PJI, 2 (2.7%) instability.
Gallart et al. (2016) [29]	Paprosky I 19 (26.4%), Paprosky IIA 12 (16.7%), Paprosky IIB 9 (12.5%), Paprosky IIC 16 (22.2%), Paprosky IIIA 12 (16.7%), Paprosky IIIB 4 (5.6%)	31 (43.1%) aseptic loosening, 27 (37.5%) PJI, 4 (5.6%) instability, 3 (4.2%) metallosis, 2 (2.8%) IMN failure, 1 (1.4%) RA, 1 (1.4%) spondyloarthritis.
Steno et al. (2015) [17]	Paprosky type I 9 (11.1%), Paprosky IIA 11 (13.6%), Paprosky IIB 27 (33.3%), Paprosky IIC 6 (7.4%), Paprosky IIIA 15 (18.5%), Paprosky IIIB 13 (16%)	66 (81.5%) aseptic loosening, 3 (3.7%) conversion hemiarthroplasty, 4 (4.9%) instability, 8 (9.9%) PJI.

**Table 6:** Comparison of components using additive manufacturing.

Manufacturer	Acetabular Cup	Composition	Tradename Porous Structure	Porosity (%)	Pore Diameter (μm)	Production Method	References:
Lima Corporate (San Daniele, Italy)	Delta TT cup	Titanium	Trabecular titanium	65	640	EBM	[37,38]
	Delta revision TT	Titanium	Trabecular titanium	65	640	EBM	[37,38]
	Delta one TT	Titanium	Trabecular titanium	65	640	EBM	[37,38]
Smith and Nephew (Memphis, TN, USA)	REDAPT	Titanium	Conceloc Advanced Porous Titanium	60–80	200–934	EBM	[14,37]
Aikang Corp. (Beijing, China)	3D ACT	Titanium	-	80	600–1000	EBM	[24]
Adler Ortho (Milan, Italy)	Omnia	Titanium	Tri-Por Cup	65–70	700	EBM	[14,37]
	Polymax ti-por	Titanium	Tri-Por Cup	65–70	700	EBM	[14,37]
	Omnia ti-por	Titanium	Tri-Por Cup	65–70	700	EBM	[14,37]
	Fixa ti-por	Titanium	Tri-Por Cup	65–70	700	EBM	[14,37]
	Agilis ti-por	Titanium	Tri-Por Cup	65–70	700	EBM	[14,37]
Stryker (Mahwah, NJ, USA)	Trident II	Titanium	Tritanium	55–65	100–700	SLM	[14,37,38]
Zimmer (Warsaw, IN, USA)	G7	Titanium	OsseoTi porous technology	70	475	-	[14,37]
Medacta (Castel San Pietro, Switzerland)	Mpact 3D metal	Titanium	3D Metal	75	600–800	EBM	[37,38]
Kyocera (Kyoto, Japan)	SQRUM TT	Titanium	-	60	640	EBM	[18,37]
Implantcast (Buxtehude, Germany)	Ecofit Epore	Titanium	EPORE	60	100–500	EBM	[14,37]
Corin (Cirencester, UK)	Trinity Plus	Titanium	PLUS (Porous layer unique structure)	50–90	300–900	-	[37]

## Data Availability

All data are available within the text of this systematic review.

## Abbreviations

The following abbreviations are used in this manuscript:

THA	Total hip arthroplasty
3-D	3-dimensional
CNC	computer numerical controlled
PRISMA	Preferred Reporting Items for Systematic reviews and meta-analyses
MINORS	Methodological Index for Non-Randomized Studies
HHS	Harris Hip Score
VAS	Visual Analogue scale
OHS	Oxford hip score
LOS	Length of stay
SF-12	Short-form 12
WOMAC	Western Ontario and McMaster Universities Osteoarthritis Index
EBM	Electron Beam Melting
SLM	Selective Laser Melting
